# Supplementary material for: Radiative cooling to deep sub-freezing temperatures through a 24-h day–night cycle
Source: Nat Commun. 2016 Dec 13;7:13729. doi: 10.1038/ncomms13729 (PMC5159822; doi:10.1038/ncomms13729)
Supplement: Supplementary Information — Supplementary Figures, Supplementary Notes, Supplementary References. [file ncomms13729-s1.pdf]

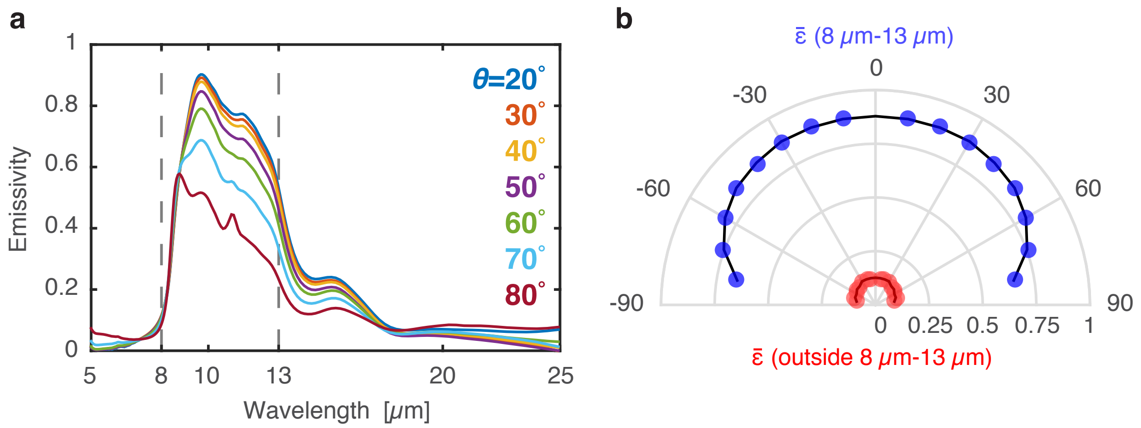

Supplementary Figure 1. **Spectral-Angular emissivity of the selective emitter. a**, Measured emissivity of the selective emitter at varying angles of incidence from 20° to 80°, with an interval of 10°, averaged over both polarizations. **b**, Average measured emissivity of the selective emitter between 8 and 13 μm is shown in blue, and average measured emissivity of the selective emitter outside the 8-13 μm atmospheric transparency window is shown in red. Both are plotted as a function of polar angle of incidence.

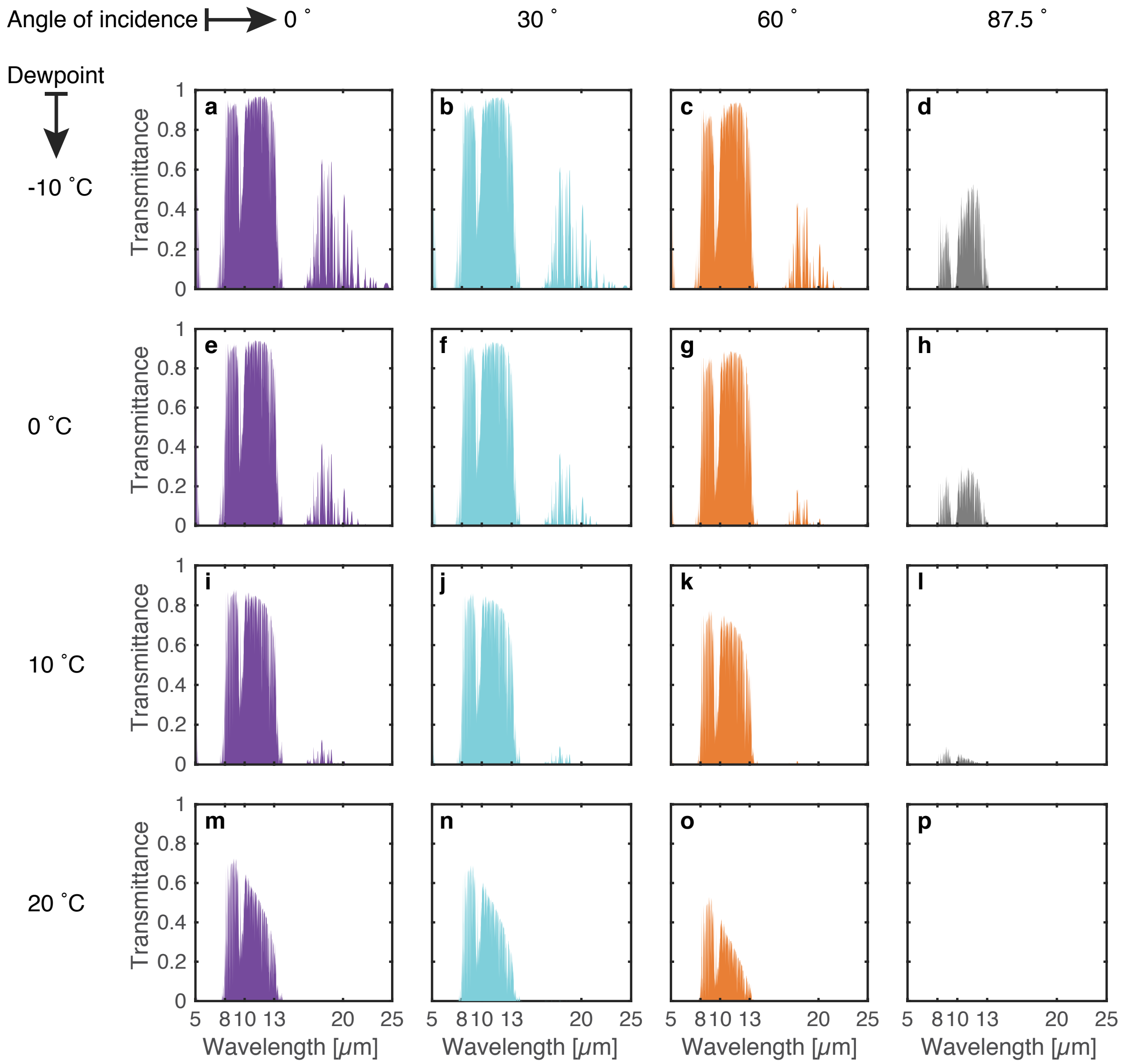

Supplementary Figure 2. The atmospheric transmittance, at varying angles of incidence and dew point temperatures. The atmospheric transmittance are obtained using ModTran5 for mid-latitude regions in winter. The atmospheric transmittance spectra are shown at 0°, 30°, 60° and 87.5° angle of incidence. **a-d**, The atmospheric transmittance at -10 ^{o}C dew point temperature. **e-h**, The atmospheric transmittance at 0 ^{o}C dew point temperature. **i-l**, The atmospheric transmittance at 10 ^{o}C dew point temperature. **m-p**, The atmospheric transmittance at 20 ^{o}C dew point temperature.

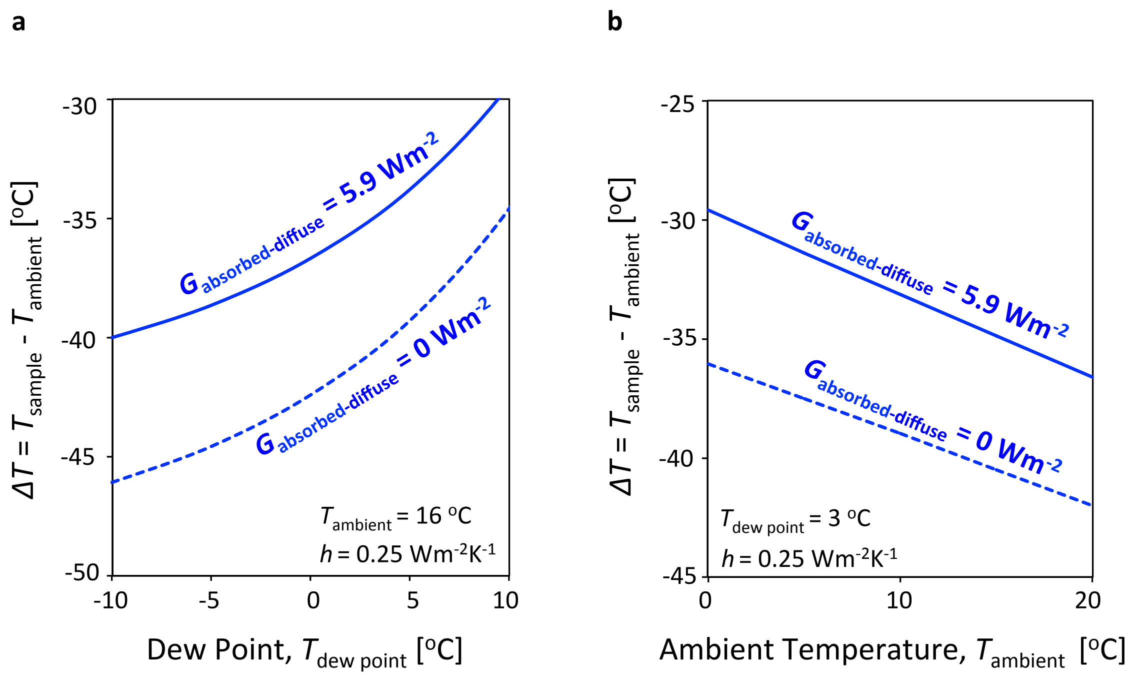

Supplementary Figure 3. Theoretical prediction of the effect of the diffuse solar irradiance on the cooling performance. The isotropic diffuse solar irradiance (solid blue line) degrades the temperature reduction by 5 °C as compared to the scenario without this component (dashed blue line). The comparison is performed in a temperature range consistent with Fig. 4(b-c) in the main text. The absorbed isotropic diffuse solar irradiance, $G_{\text{absorbed-diffuse}} = 5.9\ \text{Wm}^{-2}$, is calculated based on the measured total solar irradiance in Fig. 4a of the main text, and the model described in Supplementary Note 6.

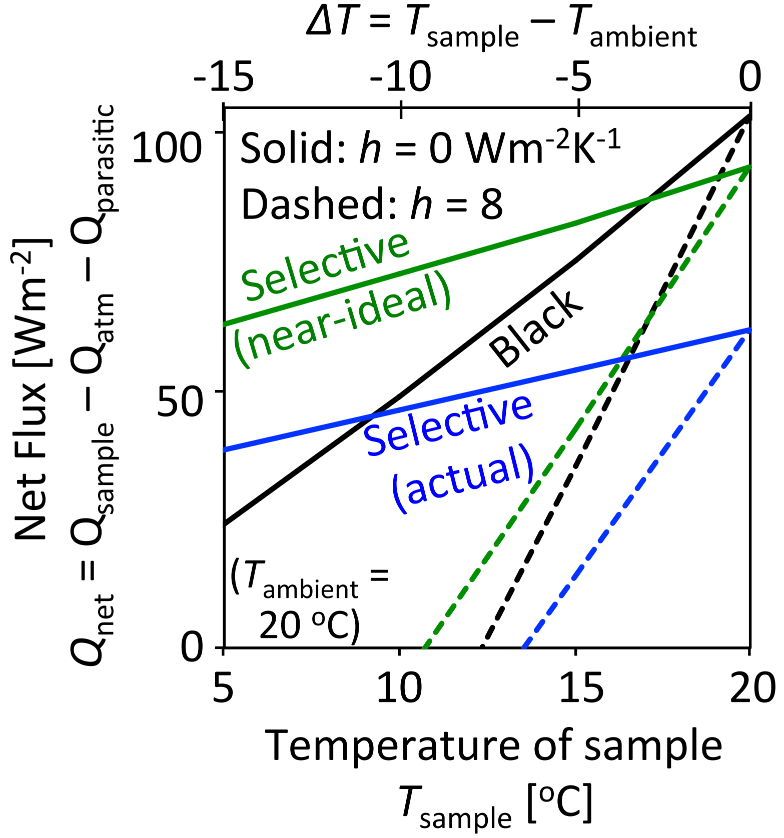

Supplementary Figure 4. **Zoom-in of Fig. 1c of the main text in the temperature range close to ambient air temperature.**

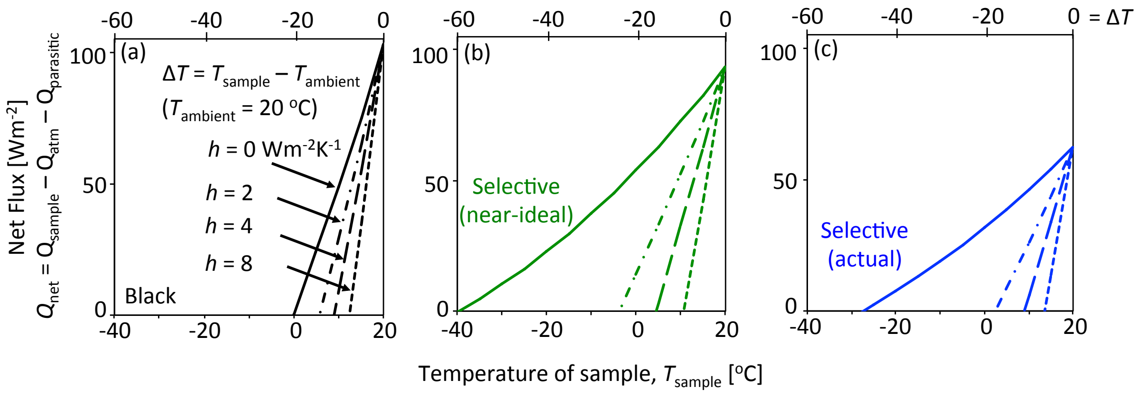

Supplementary Figure 5. **Theoretical prediction on net flux (Q_{net}) as a function of the temperature of the sample (T_{sample}), under various parasitic heat transfer coefficients (h). a**, Black emitter. **b**, Near-ideal selective emitter. **c**, Selective emitter used in this work.

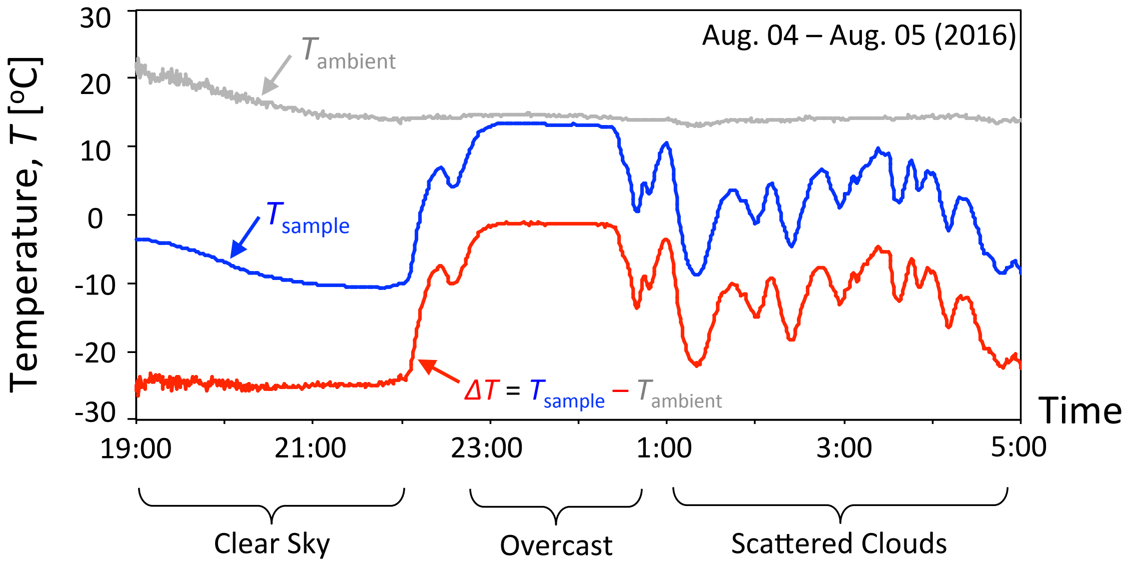

Supplementary Figure 6. **Effect of cloud coverage on the performance of radiative cooling.** Scattered clouds degrade radiative cooling, and thick and continuous clouds (overcast) nearly completely eliminates the effect radiative cooling.

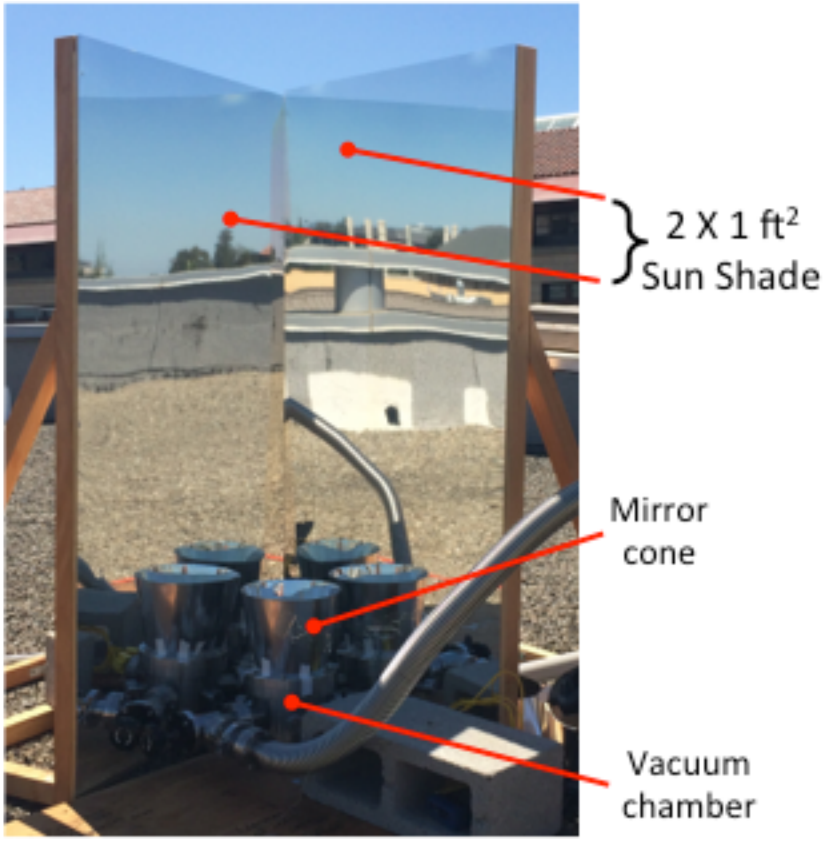

Supplementary Figure 7. **Experimental setup in summer.** The shading is more challenging in summer than in winter (see Fig. 2b in the main text), because of the high solar elevation angle (higher than 70 ^{o} at noon at Stanford), and the large range of the solar azimuthal angle (larger than 220 ^{o} from sunrise to sunset). The image here shows the experimental setup that we used in the summer which consists of two sun-shades. The setup here is able to achieve radiative cooling throughout the day in the summer without active re-positioning the shading structure under the summer sun.

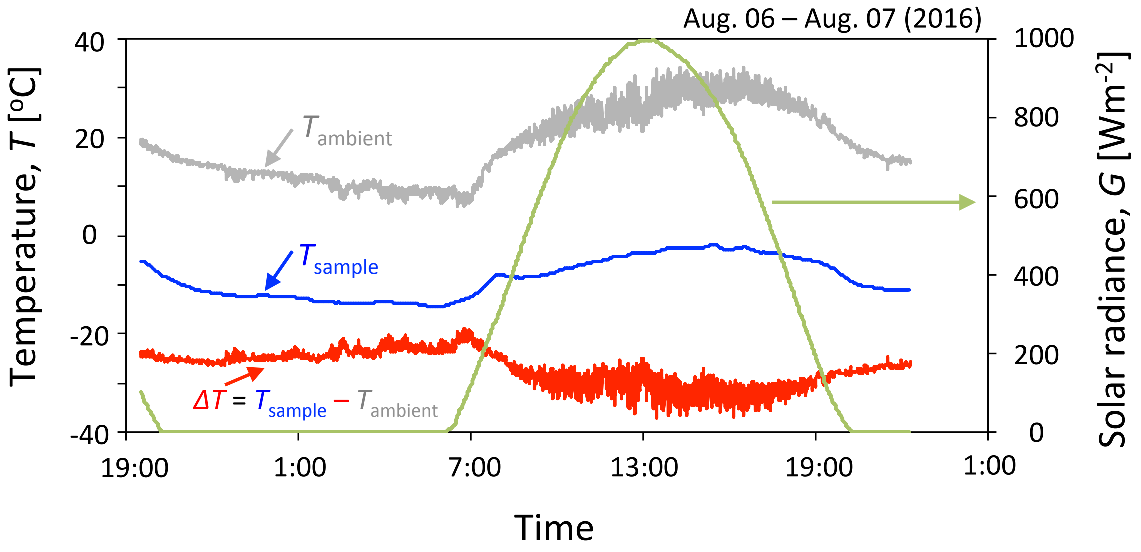

Supplementary Figure 8. 24-hour cooling results in summer. An average cooling of 27.0 ^{o}C over a 24-hour day-night cycle is achieved. The maximal cooling of 37.1 ^{o}C synchronizes with the peak of the solar irradiance, which is consistent with the winter experiment (see Fig. 4a in the main text).

Supplementary Note 1: Thermal design of the experimental apparatus

Minimizing parasitic heat losses is essential to approaching the fundamental limit of radiative cooling, as indicated in Fig. 1 of the main text. We develop an experimental apparatus (Fig. 2 of the main text) to suppress losses through all the three heat transfer modes: conduction, convection, and radiation. Following a convention in the heat transfer literature, we characterize all these heat transfer modes in terms of their effective heat transfer coefficients h. The thermal power input into the emitter is assumed to have the form $P = h \cdot A \cdot \Delta T$, where A is the area of the bottom surface of the emitter, and ΔT is the temperature difference between the emitter and the ambient. This form is valid for all these three heat transfer modes when ΔT is relatively small.

First, we conduct experiments inside high vacuum (10^{-6} Torr) to eliminate convection, and in particular to reduce air conduction. As the pressure inside the chamber decreases, the mean free path of air molecules increases, and thus the thermal conductivity of a system becomes strongly suppressed when such mean free path exceeds the characteristic length scale of the system[1]. Here we use the gap between the selective emitter and the ZnSe window as the characteristic length. As a result we obtain a parasitic heat transfer coefficient

$$h_{\mathrm{air}} = \frac{k_{\mathrm{air}}}{L_{\mathrm{gap}}}, \qquad \text{(Supplementary Equation 1)}$$

where k_{air} is the thermal conductivity of air, and $L_{\mathrm{gap}} \approx 5$ mm is the gap between selective emitter and ZnSe window. Without vacuum, $k_{\mathrm{air}} = 0.03$ $\mathrm{Wm^{-1}K^{-1}}$ at room temperature[2], and we have $h_{\mathrm{air}} = 6$ $\mathrm{Wm^{-2}K^{-1}}$. With vacuum, we calculate k_{air} based on the kinetic theory. Following the same procedure outlined in chapter 11.3.1 of Ref. 3, we estimate that k_{air} is reduced by 100, 1,000, and 10,000 times with a vacuum level of 10^{-4}, 10^{-5}, and 10^{-6} Torr, respectively. Correspondingly, h_{air} is 6×10^{-2}, 6×10^{-3}, and 6×10^{-4} $\mathrm{Wm^{-2}K^{-1}}$, respectively. Note that a vacuum level of 10^{-3} Torr, as can be achieved using a mechanical pump, does not affect too much on k_{air}, since at this vacuum level the mean-free path of air molecules is comparable to the characteristic length of the system. Thus, a mechanical pump is not adequate for our purpose. Instead, we use a turbo pump to achieve a vacuum level of 10^{-6} Torr of our vacuum chamber, and as a result the parasitic heat loss through air conduction is negligible.

Next, to reduce the radiative loss through the backside of the selective emitter, we coat its bottom surface with 150-nm-thick aluminum thin film using e-beam evaporation. In addition, we place ten concentric radiation shields[4] between the vacuum chamber floor and the selective emitter. These radiation shields are made of polished aluminum sheets

with mirror-like surfaces (Figs. 2a and 2c of the main text). We estimate the lower and upper bounds of the parasitic heat loss from backside of the selective emitter and through the concentric radiation shields. The lower bound corresponds to ideal radiation shields that only see the selective emitter but not the sky. In this ideal scenario, we have[4]

$$h_{\text{rad'n-min}} = \frac{4\varepsilon_{\text{s}}\sigma T_{\text{avg}}^{3}}{2N}, \qquad \text{(Supplementary Equation 2)}$$

where ε_{s} is the emissivity of the shields, $\sigma = 5.67\times10^{-8}$ $\text{Wm}^{-2}\text{K}^{-4}$ is the Stefan-Boltzmann constant, $N = 10$ is the number of radiation shields, and $T_{\text{avg}} = 273$ K is the average of the temperatures of the selective emitter and the chamber. The prefactor 2 in the denominator is due to the fact that each shield has two surfaces. We estimate ε_{s} to be 0.03 based on a FTIR measurement, which gives $h_{\text{rad'n-min}} = 7\times10^{-3}$ $\text{Wm}^{-2}\text{K}^{-1}$.

In our actual experimental setup, the diameter of the selective emitter is smaller than that of the shields. For a conservative estimate, we assume that the temperature of the uppermost shield to be that of the ambient, and thus we have

$$h_{rad'n-\max} = \frac{4\varepsilon_{avg}\sigma T_{avg}^{3}}{2}, \qquad \text{(Supplementary Equation 3)}$$

where ε_{avg} is the average emissivity of the shield and the aluminum thin film evaporated on the backside of the selective emitter. We use $\varepsilon_{\text{avg}} = 0.03$ since the shields are also made of shiny aluminum. In this scenario, we have $h_{\text{rad'n-max}} = 7\times10^{-2}$ $\text{Wm}^{-2}\text{K}^{-1}$.

Finally, to minimize the conductive loss, we use four hollow ceramic pegs (OMEGATITE® 200, $k_{\text{peg}} \approx 2.3\text{Wm}^{-1}\text{K}^{-1}$) to support the selective emitter above the radiation shields (Fig. 2a of the main text), and another four stainless steel threads to support the whole system above the vacuum chamber floor. We further weaken the thermal contact between the ceramic pegs and the uppermost / lowermost radiation shields by roughening the contact areas. The ten radiation shields are separated from each other by ceramic washers concentric with the ceramic pegs (Fig. 2a of the main text). Each ceramic peg has length of 0.91", and outer / inner diameters of 0.156" and 0.094", respectively. Following a similar analysis as in Supplementary Equation 1, we estimate the parasitic heat transfer coefficient through the ceramic pegs as

$$h_{\text{peg}} = \frac{k_{\text{peg}}}{L_{\text{peg}}}\cdot r, \qquad \text{(Supplementary Equation 4)}$$

where k_{peg} and L_{peg} are the thermal conductivity and length of the ceramic pegs, respectively. The parameter r is the ratio between the cross-sectional area of the four

ceramic pegs and the area of bottom surface of the selective emitter. With r = 0.2%, we have $h_{\text{pegs}} = 0.2$ Wm^{-2}K^{-1}.

With this thermal design, we estimate the parasitic heat transfer coefficient (Fig. 1a of the main text), $h = h_{\text{air}} + h_{\text{peg}} + h_{\text{rad'n}}$, to be in the range of 0.2 –0.3 Wm^{-2}K^{-1}, which, combined with the estimation of diffuse solar irradiance in Supplementary Note 5, bounds the blue shaded areas in Figs. 4b and 4c of the main text.

We also estimate the thermal time constant of the apparatus to be approximate 10 min, which is consistent with the transient behavior in Fig. 4a of the main text.

Supplementary Note 2: Fabrication and characterization of the selective emitter

The selective emitter is fabricated in Stanford Nanofabrication Facility (SNF) and Stanford Nano Shared Facilities (SNSF). We start with a 380-µm-thick, 100-mm-diameter, double-side-polished crystalline silicon wafer. During a single session of electron beam evaporation, a 150-nm-thick layer of aluminum, and a 700 -nm-thick layer of silicon, are successively evaporated on one side of the silicon wafer. A 70 -nm-thick layer of silicon nitride (Si_3N_4) is then deposited on the top by using high-density plasma chemical vapor deposition (HDPCVD). To suppress the radiative heat loss through the back side of the selective emitter, a 150-nm-thick layer of aluminum is evaporated on the other side of the silicon wafer using electron beam evaporation. The selective emitter is cleaved to fit in the vacuum chamber.

A scanning electron microscope (FEI NovaSEM 450) is used to image the selective emitter, as shown in Fig. 3a of the main text. A Fourier transform infrared (FTIR) spectrometer (Nicolet 6700, Thermo Fisher Scientific) is used to characterize the reflectance of the selective emitter with a gold film used as a reflectance standard. A variable-angle reflection accessary (Seagull, Harrick Scientific) equipped with KRS-5 substrate based wire grid polarizer (Seagull FTIR polarizer, Harrick Scientific) allows for reflectance measurements at varying angles of incidence for both polarizations. The measured spectral angular emissivity of the selective emitter is shown in Supplementary Figure 1.

In Supplementary Figure 1, we observe that the emitter exhibits strong selectivity. At 0 ˚C, the hemispherically-weighed emissivity of the emitter in the atmospheric window (8 - 13 µm) is 0.632, while that outside the atmospheric window is only 0.086. This results in

a total hemispherical emissivity of the emitter to be 0.247, since the blackbody fraction[4] of the atmospheric window at 0 ˚C is 29.5%. Such a strong selective emissivity is essential to achieving a substaintial low temperature below the ambient air temperature. In addition, the large emissivity inside the atmospheric window enables a high cooling power.

Another feature in Supplementary Figure 1 is that the emissivity of the emitter gradually decreases towards oblique angles. This is desirable for achieving ultrahigh-performance radiative cooling, as the atmosphere is increasingly opaque at larger angles of incidence (Supplementary Figure 2).

Supplementary Note 3: ZnSe window

We equip the vacuum chamber with a 4.4-inch-diameter ZnSe window (0.32 inch thick) from Laser Research Optics, as shown in Fig. 2 of the main text. The ZnSe window is double-side coated with anti-reflection layers, to enhance transmission at wavelengths centered at 10.6 μm. The transmittance of the ZnSe window are measured using Fourier transform infrared spectrometer (FTIR), as shown in Fig. 3b (red line) of the main text. Note here our FTIR is most accurate for measurement at the normal angle because of the large diameter and the large thickness of the ZnSe window. We also measured several other angles that are smaller than 45°, and found the deviation in transmittance is within 3% from that of the normal angle. Therefore, in our theoretical model below we use the results of normal angle to represent the optical properties of the ZnSe window.

Supplementary Note 4: Heat transfer model

In this section we consider nighttime cooling first. The effect of the sunlight will be treated in Supplementary Note 5. Consider a selective emitter at temperature T, with spectral angular emissivity $\varepsilon(\lambda,\Omega)$. When the selective emitter is exposed to a clear sky, it is subject to thermal radiation from the atmosphere (corresponding to the ambient air temperature $T_{ambient}$). The steady state temperature T of the selective emitter is determined by

$$Q_{\text{sample}}(T) - Q_{\text{atm}}(T_{\text{ambient}}) - Q_{\text{parasitic}} = 0 . \qquad \text{(Supplementary Equation 5)}$$

In the simple model as previously used in Ref. 5, in Supplementary Equation 5, the emitted heat flux from the selective emitter is

$$Q_{\text{sample}}(T) = \int d\Omega cos\theta \int_0^\infty d\lambda I_{\text{BB}}(T,\lambda)\varepsilon(\lambda,\Omega) . \qquad \text{(Supplementary Equation 6)}$$

Here, $\int d\Omega = \int_0^{\pi/2} d\theta sin\theta \int_0^{2\pi} d\varphi$ is an integral over the hemispherical solid angle. $I_{\text{BB}}(T,\lambda) = (4\pi\hbar c^2 / \lambda^5) / [e^{2\pi\hbar c/(\lambda k_B T)} - 1]$ is the intensity of a blackbody at temperature *T*, where $\hbar$ is the reduced Planck constant, c is the velocity of light, k_B is the Boltzmann constant, and λ is wavelength.

The absorbed heat flux from atmosphere is

$$Q_{\text{atm}}(T_{\text{ambient}}) = \int d\Omega cos\theta \int_0^{\infty} d\lambda I_{\text{BB}}(T_{\text{ambient}}, \lambda) \varepsilon(\lambda, \Omega) \varepsilon_{\text{atm}}(\lambda, \Omega). \quad \text{(Supplementary Equation 7)}$$

Here, $\varepsilon_{\text{atm}}(\lambda, \Omega)$ is the spectral angular emittance of the atmosphere. We have used Kirchhoff's law to replace absorptivity of the selective emitter with its emissivity $\varepsilon(\lambda, \Omega)$.

The parasitic heat loss is

$$Q_{\text{parasitic}} = h(T_{\text{ambient}} - T), \quad \text{(Supplementary Equation 8)}$$

where we uses an effective heat transfer coefficient, *h*, to take into account of conduction through the ceramic pegs and radiation from the back side of the selective emitter. Recall from our thermal design, we estimate *h* in the range of 0.2 -0.3 $Wm^{-2}K^{-1}$, which, combined with the estimate of diffuse solar irradiance in Supplementary Note 5, bounds the shaded bands in Figs. 4b and 4c of the main text.

In this paper, we use a more sophisticated model to take into account the effects of the ZnSe window and the mirror cone. The ZnSe window has a spectral transmittance $t_{\text{w}}(\lambda)$, reflectance $r_{\text{w}}(\lambda)$ and absorptance $\alpha_{\text{w}}(\lambda)$. Here, by energy conservation we have $t_{\text{w}}(\lambda) + r_{\text{w}}(\lambda) + \alpha_{\text{w}}(\lambda) = 1$. The ZnSe window has two effects. Since its transmissivity is less than unity, it reduces the amount of power from the atomsphere that reaches the emitter as compared to Supplementary Equation 7. On the other hand, the ZnSe window also has non-zero emissivity and part of the radiation from the window reaches the emitter. To compute the emission from the ZnSe window, we assume that the ZnSe window is at the ambient air temperature T_{ambient}. This is justified since the window is thermally very well coupled to the ambient air and the vacuum chamber.

The effect of a cone on the performance of radiative cooling has been discussed in literature[6]. The cone restricts the angular range of the apparatus to around the zenith direction where the sky is most transparent, and hence serves to prevent the relatively high intensity incoming sky radiation from the low angles from reaching the selective emitter[6]. To model the effect of the cone, we use a ray-tracing scheme to take into account the multiple reflections of photons with the cone. We use the exact geometry of the cone, including its upper and lower diameters, and its height. Due to the relatively

small size of the emitter as compared to the cone, we approximate the emitter as a point source at the center of the bottom of the cone. The cone is modeled as aluminum when calculating the spectral angular reflectivity of each reflection instance on the wall of the cone. We assume that the cone is at the ambient air temperature T_{ambient}, since it is thermally very well coupled to the ambient. This model allows us to compute the effect of the cone on both the input and output photon flux of the system.

After considering the effect of the ZnSe window and the cone, the emitted power from the selective emitter in Supplementary Equation 6 is modified to be

$$Q_{\text{sample}}(T) = \int d\Omega cos\theta \int_0^\infty d\lambda I_{\text{BB}}(T,\lambda)\varepsilon(\lambda,\Omega)\frac{t_{\text{w}}(\lambda)+\alpha_{\text{w}}(\lambda)}{1+r_{\text{w}}\left[\varepsilon(\lambda,\Omega)-1\right]}. \quad \text{(Supplementary Equation 9)}$$

Likewise, the absorbed power is modified to be

$$Q_{\text{atm}}(T_{\text{ambient}}) =$$

$$\int d\Omega cos\theta \int_0^\infty d\lambda I_{\text{BB}}(T_{\text{ambient}},\lambda)\varepsilon(\lambda,\Omega)\frac{t_{\text{w}}(\Omega)r_{\text{c}}(\Omega)\varepsilon_{\text{atm}}(\Omega_{\text{f}})+\alpha_{\text{w}}(\Omega)+t_{\text{w}}(\Omega)\left[1-r_{\text{c}}(\Omega)\right]}{1+r_{\text{w}}(\Omega)\left[\varepsilon(\Omega)-1\right]},$$

(Supplementary Equation 10)

where Ω is the direction along which a photon is emitted from the selective emitter, and Ω_{f} is the direction of the photon escaping the cone after multiple reflections. $r_{\text{c}}(\Omega)$ is the total reflectivity of a photon after sequential reflections with the cone. Here we emphasize that due to reciprocity, for any photon emitted from the selective emitter along Ω and escaped into atmosphere along Ω_{f}, there must be a corresponding photon enters the cone along Ω_{f} and finally reaches the selective emitter along Ω. These two photons have the same total reflectivity $r_{\text{c}}(\Omega)$. Supplementary Equation 10 now includes aborsorbed thermal emission from the atmosphere, the ZnSe window, and the mirror cone.

We obtain the spectral angular transmittance $t_{\text{atm}}(\lambda,\Omega)$ of the atmosphere using a standard commercial software (ModTran5)[7], at different wavelengths and incident angles. As the transparency of the atmosphere strongly depends on the amount of water vapor, we also obtain the $t_{\text{atm}}(\lambda,\Omega)$ for various dew point temperatures. The spectral angular emittance of the atmosphere is $\varepsilon_{\text{atm}}(\lambda,\Omega)=1-t_{\text{atm}}(\lambda,\Omega)$.

We show the atmospheric transmittance for varying dew point temperatures and incident angles in Supplementary Figure 2. We observe that the atmosphere has a major mid-infrared transparency window between 8-13 μm. As the dew point temperature increases, the transparency of the atmosphere decreases. For a given dew point temperature, as incident angle increases, the transparency of the atmosphere also decreases, as a result of

the longer optical path at larger incident angle. We recall from Supplementary Figure 1 that the emissivity of the selective emitter also decreases as the incident angle increases, which is a desirable feature to achieve high performance in radiative cooling.

Supplementary Note 5: Effect of the diffuse solar irradiance at daytime

Supplementary Note 4 describes a complete heat transfer model for our cooling experiment at nighttime. At daytime, one also needs to take into account the effect of sunlight. The total solar irradiance plotted in Fig. 4a of the main text contains both a direct and a diffuse component[8]. In our experimental setup, the combination of the shade and the cone blocks all direct sunlight from reaching the selective emitter. However, such a set up cannot completely eliminate diffuse sunlight from reaching the emitter. In this Section, we consider the effect of the diffuse solar irradiance. In the presence of such solar irradiance, the equation for the energy balance at steady state (Supplementary Equation 5) is modified to be

$$Q_{\text{sample}}(T) - Q_{\text{atm}}(T_{\text{ambient}}) - Q_{\text{parasitic}} - G_{\text{absorbed-diffuse}} = 0 , \qquad \text{(Supplementary Equation 11)}$$

where the new term $G_{\text{absorbed-diffuse}}$ describes the diffuse solar irradiance absorbed by the selective emitter. Below we estimate how much diffuse solar irradiance is absorbed by the selective emitter.

We first deduce the total diffuse solar irradiance based on our measured total solar irradiance. According to the Erbs correlation (Eq. 2.10.1 of Ref. 8), for a clear sky we have

$$G_{\text{diffuse}} = 16.5\% \cdot G_{\text{total}} , \qquad \text{(Supplementary Equation 12)}$$

where the total solar irradiance G_{total} is the sum of the diffuse (G_{diffuse}) and the direct (G_{direct}) components.

There are four components of the total diffuse solar irradiance[9]: an isotropic diffuse component (uniform from the sky dome), circumsolar diffuse component (resulting from the forward scattering of solar radiation and concentrated in an area close to the sun), horizon brightening component (concentrated in a band near the horizon), and a reflected component that accounts for the radiation reflected from the ground to the selective emitter. For our experimental setup, a horizontal selective emitter with sun-shade and mirror-cone, only the isotropic diffuse component can reach the selective emitter.

We next estimate the portion of this isotropic diffuse component among the total diffuse solar irradiance. Based on the HDKR model (Eq. 2.16.7 of Ref. 8), this isotropic portion is

$1 - A_i$, (Supplementary Equation 13)

where A_i is the anisotropy portion (Eq. 2.16.3 of Ref. 8), which can be expressed as

$$A_i = \left(1 - \frac{G_{diffuse}}{G_{total}}\right) K_T, \quad \text{(Supplementary Equation 14)}$$

where K_T is the clearness index (Eq. 2.9.3 of Ref. 8). A clear sky (high K_T) results in a high A_i, which implies that the anisotropic component dominates the total diffuse solar irradiance.

To estimate the upper bound of the isotropic portion, in the Erbs correction (Eq. 2.10.1 of Ref. 8), we choose a lower bound of K_T (=0.8) under the condition of a clear sky, which results in an anisotropy portion of 66.8% and an isotropic portion of 33.2%.

We finally quantify the portion of this isotropic diffuse component that is absorbed by the selective emitter. In our model, we calculate the angular spectral absorptivity of the selective emitter, and the angular spectral transmissivity and reflectivity of the ZnSe window, over wavelength range of solar irradiance. We also take into account the real geometry of the mirror-cone. Assuming that the isotropic diffuse solar irradiance has the same spectrum shape as the AM1.5 solar spectrum, 21.1% of the isotropic diffuse component is expected to be absorbed by the selective emitter tested in this work.

To summarize, for the total solar irradiance measured in Fig. 4a of the main text, 16.5% is diffuse component, in which a maximal portion of 33.2% is isotropic. Finally, only 21.1% of this isotropic diffuse solar irradiance is absorbed by the selective emitter. Thus, for a peak total solar irradiance of 512 Wm^{-2} as measured in Fig. 4a of the main text, the maximal isotropic diffuse solar irradiance absorbed by the selective emitter is 512 × 16.5% × 33.2% × 21.1% = 5.9 Wm^{-2}.

Supplementary Figure 3 illustrates the effect of the diffuse solar irradiance. The cooling performance taking into account the 5.9 Wm^{-2} diffuse solar irradiance absorbed by the selective emitter (blue solid line) is degraded by 5-6 K as compared to the scenario without the diffuse solar component (blue dashed line). Here we fix the ambient temperature to be $T_{ambient}$ = 16 ^oC in Supplementary Figure 3a, and the dew point to be $T_{dew\ point}$ = 3 ^oC in Supplementary Figure 3b, which is consistent with Fig. 4b and Fig. 4c of the main text, respectively. We use a parasitic heat transfer coefficient h = 0.25$Wm^{-2}K^{-1}$ which is an average of the upper and lower bounds estimated based on our thermal design in Supplementary Note 1.

Note that the experimental data in Fig. 4b-c of the main text are a mixture of daytime and nighttime measurements. Also recall from our thermal design, we estimate the parasitic heat transfer coefficient, h, in the range of 0.2 -0.3 $Wm^{-2}K^{-1}$. As a result, in the shaded bands in Fig. 4b and 4c of the main text, we set $h = 0.2\ Wm^{-2}K^{-1}$, and a diffuse solar irradiance, $G_{absorbed\text{-}diffuse} = 0\ Wm^{-2}$, for the lower bound, and $h = 0.3\ Wm^{-2}K^{-1}$ and $G_{absorbed\text{-}diffuse} = 5.9\ Wm^{-2}$, for the upper bound.

Supplementary Note 6: The use of selective emitters in applications with a cooling load

The selective emitter used in this work is designed to approach the fundamental limit of temperature reduction of radiative cooling. Applications demanding high cooling load would instead operate in a temperature range closer to the ambient air temperature, with the aim of achieving a higher cooling power in this temperature range. Therefore, in Supplementary Figure 4, we replot Fig. 1c in the main text in the temperature range between 5-20°C when the ambient air temperature is at 20°C.

As shown in Supplementary Figure 4, in a practical scenario of a parasitic heat transfer coefficient $h = 8\ Wm^{-2}K^{-1}$, the cooling power of the selective emitter (dashed blue line) used in this work is lower than that of a black emitter (dashed black line) in the entire temperature range. This arises primarily because the emissivity of the selective emitter used in the experiments is significantly lower than unity in the transparency window. On the other hand, a near-ideal selective emitter (dashed green line) has a higher cooling power as compared to the black emitter, as long as the temperature of the cooler is maintained at more than 3°C below ambient.

Supplementary Note 7: Extending Fig. 1c of the main text to include more parasitic heat transfer coefficients

For visualization of gradual improvements on the cooling performance by gradually decreasing parasitic heat transfer coefficients, in Supplementary Figure 5 we include more h values.

Supplementary Note 8: Radiative cooling under cloudy sky

Intuitively, the cloud closes the atmospheric transparency window, and thus degrade or totally eliminate the effect of radiative cooling. This effect of cloud coverage has been well documented in literature[10,11].

Supplementary Figure 6 shows the contrast on the performance of radiative cooling under a clear sky vs. a cloudy sky. While scattered clouds degrade the temperature reduction, thick and continuous clouds (overcast) completely suppresses the effect of radiative cooling.

Supplementary Note 9: Cooling experiments in summer

The cooling experiments in summer is more challenging because of the difficulty in shading. At Stanford in summer, the solar elevation angle could reach beyond 70°, while it is below 40° in winter[12]. Moreover, the range of the azimuthal angle in summer is larger than 220° from sunrise to sunset[12]. These two facts make the shading of the experimental setup difficult.

Nevertheless, with some revision the same strategy of using a combination of solar shade and cone can be applied in the summer time as well. As shown in Supplementary Figure 7, we fix two 1 by 2 ft^2 shiny metal sheets at a specific position to shade the chamber from sunrise to sunset. We emphasize that in a 24-hour day-night cycle, we do not adjust the position of the shading structure.

Supplementary Figure 8 shows the experimental results of a 24-hour day-night cooling in summer. An average cooling of 27.0 ^oC is achieved with the shading scheme in Supplementary Figure 7. The maximal cooling of 37.1 ^oC also synchronizes with the peak of the solar irradiance, which is consistent with the winter experiment in Fig. 4a of the main text.

Supplementary References

1. Springer, G. S. in *Advances in Heat Transfer*, Vol. 7 (eds. Irvine, T. F. & Hartnett, J. P.) Pages 163-218 (Elsevier, 1971).
2. Lienhard, J. H. & Lienhard, J. H. *A Heat Transfer Textbook*. (Phlogiston Press, 2012).
3. Dames, C, in *Heat Conduction*, 3rd edn, (lead author Jiji, L. M.), Ch. 11 (Springer, 2009).
4. Siegel, R. & Howell, J. R. *Thermal Radiation Heat Transfer*. (Taylor & Francis, 1992).
5. Raman, A. P., Anoma, M. A., Zhu, L., Rephaeli, E. & Fan, S. Passive radiative cooling below ambient air temperature under direct sunlight. *Nature* **515,** 540–544 (2014).
6. Smith, G. B. Amplified radiative cooling via optimised combinations of aperture geometry and spectral emittance profiles of surfaces and the atmosphere. *Sol. Energy Mater. Sol. Cells* **93,** 1696–1701 (2009).
7. Berk, A. *et al.* ModTran 5: 2006 update. in *Proc SPIE Int Soc Opt Eng* **6233,** 62331F–62331F–8 (2006).
8. Duffie, J. A. & Beckman, W. A. *Solar Engineering of Thermal Processes*. (John Wiley & Sons, 2013).
9. Loutzenhiser, P. G. *et al.* Empirical validation of models to compute solar irradiance on inclined surfaces for building energy simulation. *Sol. Energy* **81,** 254–267 (2007).
10. Castro Aguilar, J. L., Gentle, A. R., Smith, G. B. & Chen, D. A method to measure total atmospheric long-wave down-welling radiation using a low cost infrared thermometer tilted to the vertical. *Energy* **81,** 233–244 (2015).
11. Gentle, A. & Smith, P. G. Performance comparisons of sky window spectral selective and high emittance radiant cooling systems under varying atmospheric conditions . in *Sol. 2010 Proc. 48th AuSES Annu. Conf.* 1–8 (2011).
12. http://www.esrl.noaa.gov/gmd/grad/solcalc/azel.html.
